# Fibronectin fragment-induced expression of matrix metalloproteinases is mediated by MyD88-dependent TLR-2 signaling pathway in human chondrocytes

**DOI:** 10.1186/s13075-015-0833-9

**Published:** 2015-11-12

**Authors:** Hyun Sook Hwang, Su Jin Park, Eun Jeong Cheon, Mi Hyun Lee, Hyun Ah Kim

**Affiliations:** Division of rheumatology, Department of Internal Medicine, Hallym University Sacred Heart Hospital, 896, Pyungchon, Anyang, Kyunggi 431-070 Korea; Institute for Skeletal Aging, Hallym University, Chunchon, 200-702 Korea

**Keywords:** Chondrocyte, Fibronectin fragment, MyD88, Osteoarthritis, TLR-2

## Abstract

**Introduction:**

Fibronectin fragments (FN-fs) are increased in the cartilage of patients with osteoarthritis (OA) and have a potent chondrolytic effect. However, little is known about the cellular receptors and signaling mechanisms that are mediated by FN-fs. We investigated whether the 29-kDa amino-terminal fibronectin fragment (29-kDa FN-f) regulates cartilage catabolism via the Toll-like receptor (TLR)-2 signaling pathway in human chondrocytes.

**Methods:**

Small interfering RNA was used to knock down TLR-2 and myeloid differentiation factor 88 (MyD88). TLR-2 was overexpressed in chondrocytes transfected with a TLR-2 expression plasmid. The expression levels of matrix metalloproteinase (MMP)-1, MMP-3, and MMP-13 were analyzed using quantitative real-time reverse transcription polymerase chain reactions, immunoblotting, or enzyme-linked immunosorbent assay. The effect of TLR-2 on 29-kDa FN-f-mediated signaling pathways was investigated by immunoblotting.

**Results:**

TLR-2, TLR-3, TLR-4, and TLR-5 mRNA were significantly overexpressed in OA cartilage compared with normal cartilage, whereas no significant difference of TLR-1 mRNA expression was found. 29-kDa FN-f significantly increased TLR-2 expression in human chondrocytes in a dose- and time-dependent manner. Knockdown of TLR-2 or MyD88, the latter a downstream adaptor of TLR-2, significantly inhibited 29-kDa FN-f-induced MMP production at the mRNA and protein levels. Conversely, TLR-2 overexpression led to enhanced MMP production by 29-kDa FN-f. In addition, TLR-2 knockdown apparently inhibited 29-kDa FN-f-mediated activation of phosphorylated nuclear factor of kappa light polypeptide gene enhancer in B-cells inhibitor, alpha, and p38, but not of c-Jun N-terminal kinase or extracellular signal-regulated kinase. Exposure to synovial fluid (SF) from affected joints of patients with OA elevated MMP-1, MMP-3, and MMP-13 expression markedly in primary chondrocytes without reducing cell viability. However, TLR-2 knockdown in chondrocytes significantly suppressed SF-induced MMP induction.

**Conclusions:**

Our data demonstrate that the MyD88-dependent TLR-2 signaling pathway may be responsible for 29-kDa FN-f-mediated cartilage catabolic responses. Our results will enhance understanding of cartilage catabolic mechanisms driven by cartilage degradation products, including FN-f. The modulation of TLR-2 signaling activated by damage-associated molecular patterns, including 29-kDa FN-f, is a potential therapeutic strategy for the prevention of cartilage degradation in OA.

**Electronic supplementary material:**

The online version of this article (doi:10.1186/s13075-015-0833-9) contains supplementary material, which is available to authorized users.

## Introduction

Articular cartilage contains abundant extracellular matrix (ECM), the degradation of which is a central event leading to joint destruction in many arthritic conditions, including rheumatoid arthritis (RA), osteoarthritis (OA), and septic arthritis [1]. Chondrocytes respond to a variety of stimuli, including proinflammatory cytokines and mechanical loading, by expressing degradative enzymes and catabolic mediators [1, 2]. Fibronectin (FN) is an ECM glycoprotein found in body fluids and many cell types, and an increase in its expression is associated with tissue remodeling and repair [3, 4]. In normal cartilage, FN is localized mainly in the matrix of the surface zone. However, in OA, there is an up to tenfold FN accumulation in cartilage, equivalent to approximately 15 μg/ml wet weight [5]. This results from an increase in both FN synthesis and accumulation and in FN binding in the lesion area [6, 7].

In addition, elevated levels of fibronectin fragments (FN-fs) are found in the OA joint milieu [8]. It has been estimated that up to 50 % of the FN in arthritic synovial fluid (SF) is fragmented into molecular sizes of 29–200 kDa [9]. In OA SF, FN-fs can attain levels greater than 1 μM due to a combination of increased FN production and proteolytic enzyme activation [7]. In particular, the most potent fragment, 29-kDa amino-terminal fibronectin fragment (29-kDa FN-f), stimulates proteoglycan breakdown by enhancing nitric oxide (NO) production [10] and increases the expression levels of matrix metalloproteinase (MMP)-1, MMP-3, and MMP-13 [11]. However, little is known about how the FN-fs mediate catabolic signaling in chondrocytes.

Toll-like receptors (TLRs) have been implicated in various cellular responses, including innate immunity, a host defense system against invading pathogens, as well as in autoimmune and inflammatory diseases [12]. TLRs are pattern recognition receptors that recognize pathogen-associated molecular patterns, including bacterial peptidoglycan (PGN) and lipopolysaccharide (LPS), and damage-associated molecular patterns (DAMPs) released upon tissue injury [13]. The treatment of chondrocytes using *Staphylococcus aureus* PGN and LPS significantly increased TLR-2 mRNA expression and the production of MMPs, NO, and prostaglandin E_2_ [2]. In addition, microcrystals, including calcium pyrophosphate dihydrate and monosodium urate, deposited in the synovium and articular cartilage stimulate NO production via TLR-2-mediated signaling [14].

In the present study, we found that 29-kDa FN-f-induced catabolic responses were regulated via the myeloid differentiation factor 88 (MyD88)-dependent TLR-2 signaling pathway in human articular chondrocytes. Our results provide new insight into the regulatory mechanism for FN-f-triggered catabolic responses via TLR-2 and suggest that targeting TLR-2 may have positive therapeutic effects in FN-f-aggravated OA cartilage damage.

## Methods

### Materials and antibodies

Human plasma FN and 120-kDa FN-f were purchased from EMD Millipore (Temecula, CA, USA). The N-terminal 45-kDa FN-f and 29-kDa FN-f were purchased from Sigma-Aldrich (St. Louis, MO, USA). Endotoxin contained in all reagents, including FN, FN-fs, and SF, was quantified using a Pierce LAL Chromogenic Endotoxin Quantitation Kit (Thermo Scientific, Rockford, IL, USA), and we confirmed that the reagents were endotoxin-free. Antibodies to MMP-1, MMP-3, and β-actin were obtained from R&D Systems (Minneapolis, MN, USA). Antibodies to phosphorylated c-Jun N-terminal kinase (p-JNK), phosphorylated p38 (p-p38), phosphorylated nuclear factor of kappa light polypeptide gene enhancer in B-cells inhibitor, alpha (p-IκBα), and phosphorylated extracellular signal-regulated kinase (p-ERK) were purchased from Cell Signaling Technology (Danvers, MA, USA). Horseradish peroxidase-conjugated secondary antibodies were obtained from Santa Cruz Biotechnology (Santa Cruz, CA, USA).

### Patients

Cartilage samples were obtained at the time of total knee replacement surgery from OA patients [n = 20, mean ± standard deviation (SD) age 71.5 ± 7.3 years] who were diagnosed according to the American College of Rheumatology criteria [15, 16]. Normal cartilage samples were obtained from patients with femoral neck fractures with no known history of OA or RA (n = 15, mean ± SD age 69.8 ± 11.2 years). The collection and use of human tissue samples were reviewed and approved by the institutional review board of Hallym University Sacred Heart Hospital (Anyang, Korea; approval number 2013-I022). All patients provided written informed consent.

### Cell culture

We isolated primary human chondrocytes from articular cartilage that was dissected and subjected to sequential digestion using protease from *Streptomyces griseus* (Sigma-Aldrich), collagenase from *Clostridium histolyticum* (Sigma-Aldrich), and hyaluronidase from bovine testes (Sigma-Aldrich) in Dulbecco’s modified Eagle’s medium (DMEM; Life Technologies, Frederick, MD, USA). Chondrocytes were maintained in DMEM containing 10 % fetal bovine serum and 1 % penicillin-streptomycin. The cells were incubated at 37 °C in a humidified atmosphere of 5 % CO_2_, and the medium was changed every 2–3 days. First-passage cultured human chondrocytes were used for all experiments within 3–6 days after seeding.

### Transfection of small interfering RNAs and plasmids

TLR-2, MyD88, and control small interfering RNA (siRNA) were purchased from Bioneer (Daejeon, Korea). A mammalian expression plasmid containing human TLR-2 and empty vector were obtained from Addgene (Cambridge, MA, USA). The effects of siRNA and the expression plasmid were monitored using quantitative real-time reverse transcription polymerase chain reactions (qRT-PCRs). Human chondrocytes were transfected with 50 nM siRNA or 0.5 μg of expression plasmid using the calcium phosphate precipitation method [17]. Forty-eight hours after transfection, the cells were stimulated with 29-kDa FN-f for the indicated time periods and used for the following experiments. Transfection of small interfering Toll-like receptor 2 (siTLR-2) did not lead to higher cell death than control siRNA transfection as assessed by 3-(4,5-dimethylthiazol-2-yl)-2,5-diphenyltetrazolium bromide assay.

### qRT-PCR analysis

Total RNA was extracted using a standard protocol with TRIzol reagent (Invitrogen/Thermo Fisher Scientific, Carlsbad, CA, USA). In some studies, cartilage tissue from the knees of normal and OA donors was crushed to a fine powder in liquid nitrogen, and total RNA was prepared as described above. The first-strand cDNA was synthesized from 2 μg of total RNA, using Moloney murine leukemia virus reverse transcriptase (Promega, Madison, WI, USA). The PCRs were performed using the LightCycler FastStart DNA Master SYBR Green I kit (Roche Diagnostics, Mannheim, Germany) and a LightCycler 2.0 Instrument (Roche Diagnostics, Indianapolis, IN, USA). Glyceraldehyde 3-phosphate dehydrogenase (GAPDH) was used as a reference gene. Primer sequences were as follows: TLR-1 forward 5′-AGT TGT CAG CGA TGT GTT CG-3′, reverse 5′-AAA ATC CAA ATG CAG GAA CG-3′; TLR-2 forward 5′-CCT CCA ATC AGG CTT CTC TG-3′, reverse 5′-TGG AGG TTC ACA CAC CTC TG-3′; TLR-3 forward 5′-AGC CTT CAA CGA CTG ATG CT-3′, reverse 5′-TTT CCA GAG CCG TGC TAA GT-3′; TLR-4 forward 5′-TGA GCA GTC GTG CTG GTA TC-3′, reverse 5′-CAG GGC TTT TCT GAG TCG TC-3′; TLR-5 forward 5′-GGA ACC AGC TCC TAG CTC CT-3′, reverse 5′-AAG AGG GAA ACC CCA GAG AA-3′; MyD88 forward 5′-GCA CAT GGG CAC ATA CAG AC-3′, reverse 5′-GAC ATG GTT AGG CTC CCT CA-3′; MMP-1 forward 5′-AGT GAC TGG GAA ACC AGA TGC TGA-3′, reverse 5′-GCT CTT GGC AAA TCT GGC GTG TAA-3′; MMP-3 forward 5′-GCG TGG ATG CCG CAT ATG AAG TTA-3′, reverse 5′-AAA CCT AGG GTG TGG ATG CCT CTT-3′; MMP-13 forward 5′-AAG GAC CCT GGA GCA CTC ATG TTT-3′, reverse 5′-TGG CAT CAA GGG ATA AGG AAG GGT-3′; GAPDH forward 5′-TGA TGA CAT CAA GAA GGT GGT GAA G-3′, reverse 5′-TCC TTG GAG GCC ATG TGG GCC AT-3′.

### Western blot analysis

Following 29-kDa FN-f stimulation, culture supernatants were collected, and the cells were washed with cold phosphate-buffered saline and lysed in lysis buffer [50 mM sodium acetate, pH 5.8, 10 % sodium dodecyl sulfate (SDS), 1 mM ethylenediaminetetraacetic acid, 1 mM phenylmethylsulfonyl fluoride, and 1 μg/ml aprotinin] at 4 °C. Proteins were subjected to 10 % SDS-polyacrylamide gel electrophoresis and transferred to polyvinylidene difluoride membranes (EMD Millipore). After blocking with 5 % nonfat milk in Tris-buffered saline plus 0.1 % Tween 20, the membranes were incubated with primary antibodies to MMP-1, MMP-3, p-JNK, p-p38, p-IκBα, p-ERK, or β-actin. The membrane was developed using an enhanced chemiluminescence kit (Santa Cruz Biotechnology).

### MMP-13 enzyme-linked immunosorbent assay

The human chondrocyte culture supernatants were harvested following 29-kDa FN-f stimulation for 24 h, and the MMP-13 protein level was determined by enzyme-linked immunosorbent assay (ELISA) using a pro-MMP-13 immunoassay kit according to the manufacturer’s instructions (R&D Systems). Plates were read at 450 nm using a Thermo Scientific Multiskan GO Microplate Spectrophotometer (Thermo Fisher Scientific, Vantaa, Finland).

### Preparation of synovial fluid samples

SF was obtained from patients with OA [n = 4, age (mean ± SD) 73.0 ± 14.7 years] and centrifuged at 14,000 rpm for 10 minutes to remove cells and joint debris. The SF supernatants were stored at −80 °C until used for assays. Chondrocytes transfected with TLR-2 or control siRNA were exposed to SF diluted with serum-free DMEM for 24 h, washed twice with serum-free DMEM, and incubated in serum-free DMEM for 24 h. The levels of MMP-1 and MMP-3 released from the chondrocytes were determined using Western blot analysis and that for MMP-13 was determined by ELISA.

### Statistical analysis

The results are expressed as means ± SD. Statistical analysis was performed using the Mann–Whitney *U* test or two-way analysis of variance. *P* < 0.05 was taken to indicate statistical significance. All experiments were repeated at least in triplicate, and representative data are presented.

## Results

### Differential expression of TLR family members in normal and OA cartilage and fibronectin fragment-regulated TLR-2 expression

**Fig. 1:**
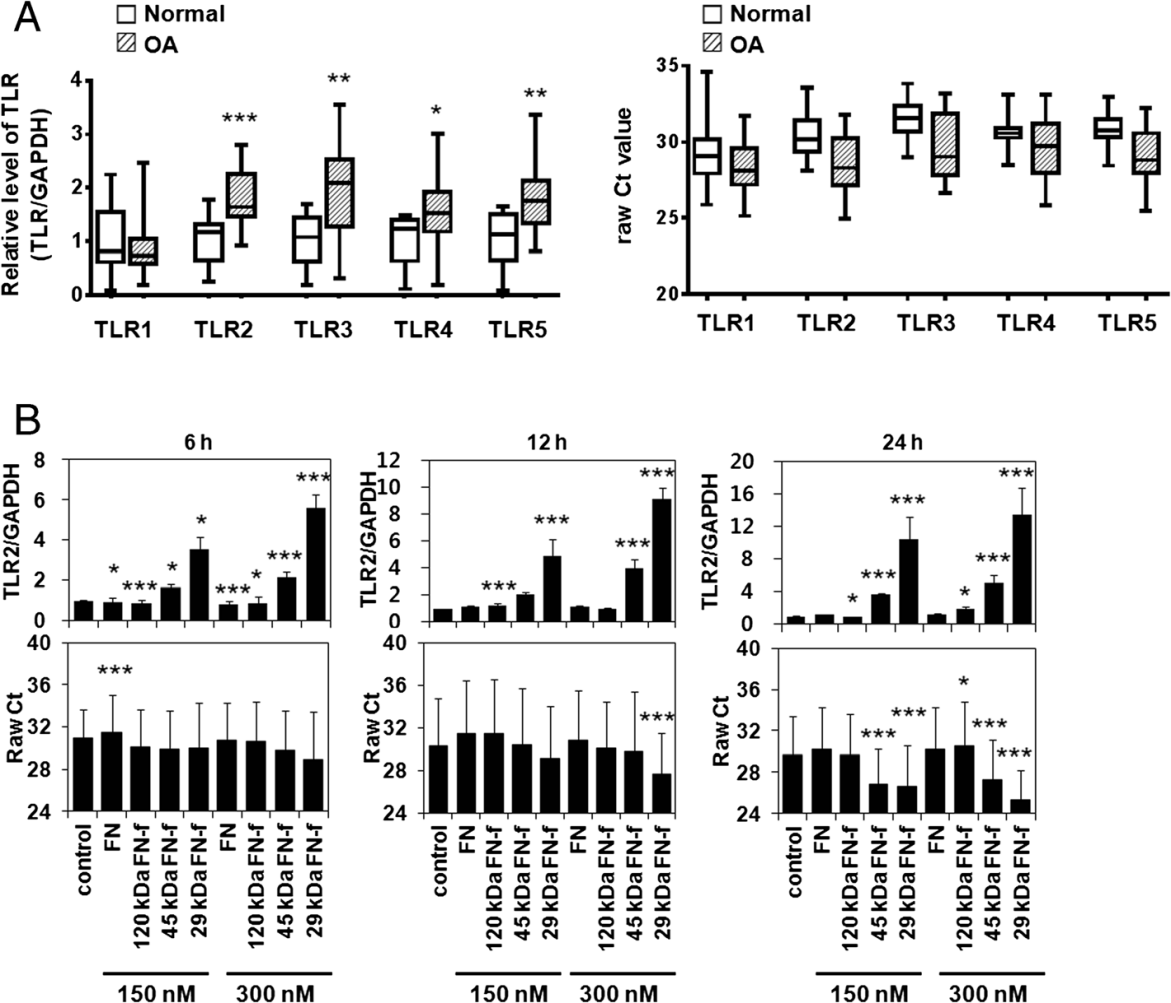
Differential expression of Toll-like receptor (TLR) family members in normal and osteoarthritic (OA) cartilage and fibronectin fragment (FN-f)-regulated TLR-2 expression in human chondrocytes. **a** Expression of the TLR-1–TLR-5 genes in normal and OA cartilage was determined using SYBR Green-based real-time polymerase chain reaction. The expression level of TLR family members was normalized to that of glyceraldehyde 3-phosphate dehydrogenase (GAPDH). Cycle threshold changes (Δ*C*_t_) were calculated by subtracting *C*_t_ values of GAPDH from *C*_t_ values of TLR family members. The mean value of gene expression level in normal cartilage was designated as 1 in each experiment. Also, the expression levels of TLR family members were described as raw *C*_t_ values. Data represent the mean ± SD for duplicate experiments from ten separate donors. Lines within the boxes represent the median, boxes represent the 25th and 75th percentiles, and lines outside the boxes correspond to the minimum and maximum values. **b** TLR-2 mRNA expression levels in OA chondrocytes were measured at 6, 12, and 24 h after treatment with intact FN or various FN-fs. The expression level of TLR-2 was described as raw *C*_t_ values as well as relative values, normalized to that of GAPDH. Data represent the mean ± SD for triplicate experiments from three different donors (n = 3). *P < 0.05, **P < 0.01, and ***P < 0.001 vs*.* control

On the basis of previous reports on the differential expression of specific TLR family members in human articular chondrocytes from normal and OA cartilage [2, 18], we investigated differences in the expression of the TLR-1–TLR-5 genes in normal and OA cartilage. qRT-PCR analysis showed that the TLR-1–TLR-5 genes were expressed in both normal and OA cartilage (Fig. 1a). Although basal expression levels varied from individual to individual, TLR-2, TLR-3, TLR-4, and TLR-5 expression levels were significantly elevated in OA cartilage compared with normal cartilage. In contrast, the TLR-1 expression levels did not differ between normal and OA cartilage (Fig. 1a). Next, we decided to determine whether TLR-2 expression can be regulated by potential endogenous ligand FN-fs. TLR-2 expression in human chondrocytes was significantly enhanced by 45-kDa and 29-kDa FN-fs in a time- and dose-dependent manner (Fig. 1b). In particular, 29-kDa FN-f induced the highest level of TLR-2 expression, whereas intact FN did not change TLR-2 expression, and 120-kDa FN-f (300 nM) significantly increased TLR-2 expression only at 24 h compared with untreated human chondrocytes (Fig. 1b). 29-kDa FN-f slightly but significantly increased TLR-1, TLR-3, and TLR-4 expression levels after 6 h of stimulation (Additional file 1). These results suggest that the increased TLR-2 expression in OA cartilage may be related to FN-f products generated by cartilage destruction.

### 29-kDa FN-f stimulates TLR-2-dependent procatabolic responses in human articular chondrocytes

**Fig. 2:**
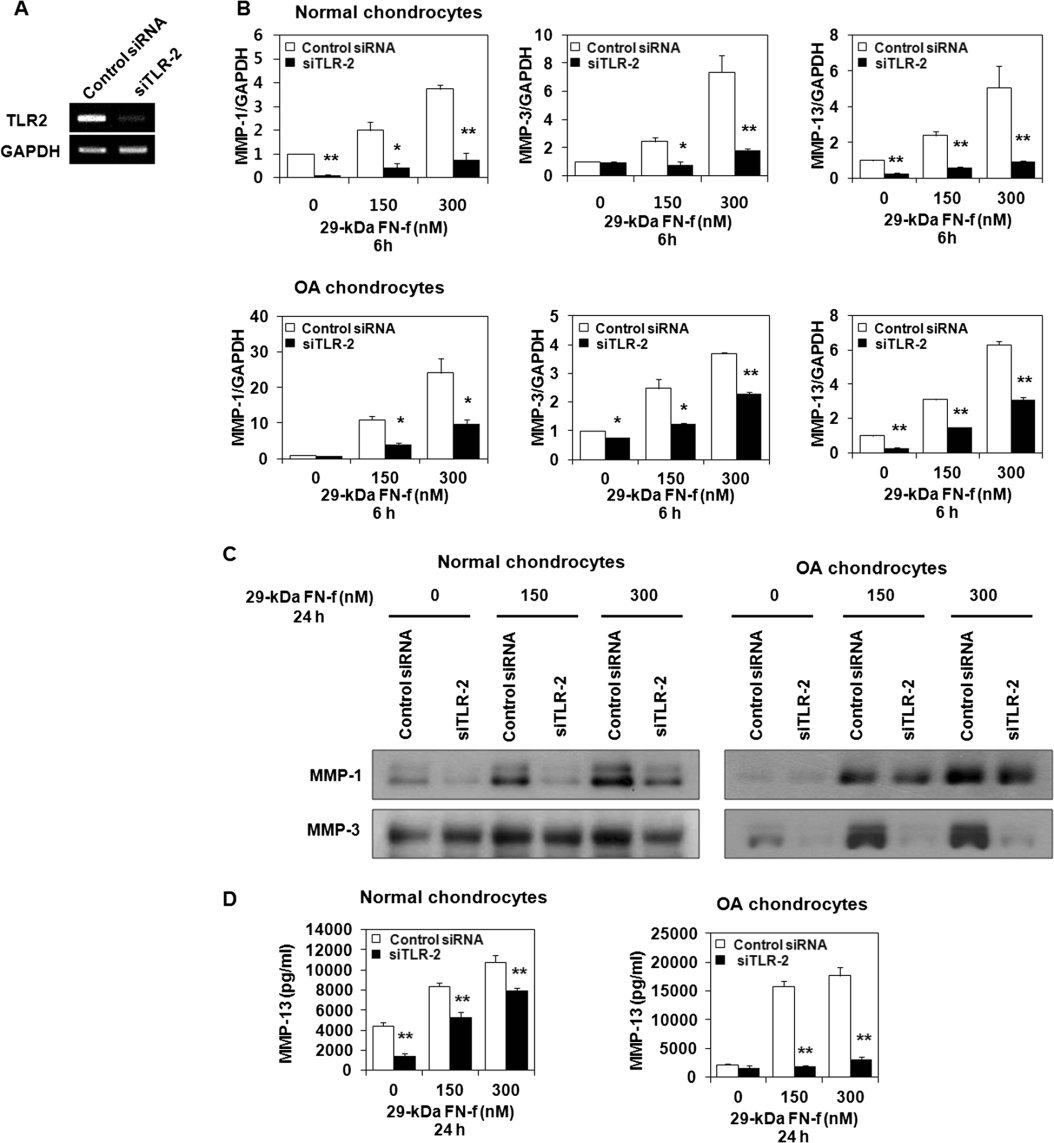
Small interfering Toll-like receptor 2 (siTLR-2) knockdown of endogenous TLR-2 expression suppresses 29-kDa amino-terminal fibronectin fragment (29-kDa FN-f)-induced matrix metalloproteinase (MMP) production in normal and osteoarthritis (OA) chondrocytes. Chondrocytes were transfected with siTLR-2 or control small interfering RNA (siRNA). Cells were stimulated 48 h after transfection using different concentrations of 29-kDa FN-f for 6 or 24 h. **a** Real-time reverse transcription polymerase chain reaction (RT-PCR) analysis demonstrated specific inhibition of TLR-2 expression by siRNA against TLR-2. **b** In normal and OA chondrocytes, MMP-1, MMP-3, and MMP-13 mRNA expression was analyzed using SYBR Green RT-PCR. Glyceraldehyde 3-phosphate dehydrogenase (GAPDH) was used as an internal control. The mRNA expression level in untreated control siRNA transfected chondrocytes was set as 1. Data represent the mean ± SD for triplicate experiments from three different donors. *P < 0.05, **P < 0.01, and ***P < 0.001 vs*.* control. **c** Secretion of MMP-1 and MMP-3 in supernatants of 29-kDa FN-f-stimulated chondrocytes was assessed using Western blot analysis. Data are representative of four independent experiments from different donors with similar results. **d** MMP-13 production in culture supernatants was determined by enzyme-linked immunosorbent assay. Each value represents the mean ± SD of triplicate experiments from three different donors. *P < 0.05 and **P < 0.01 vs*.* control siRNA-transfected chondrocytes

**Fig. 3:**
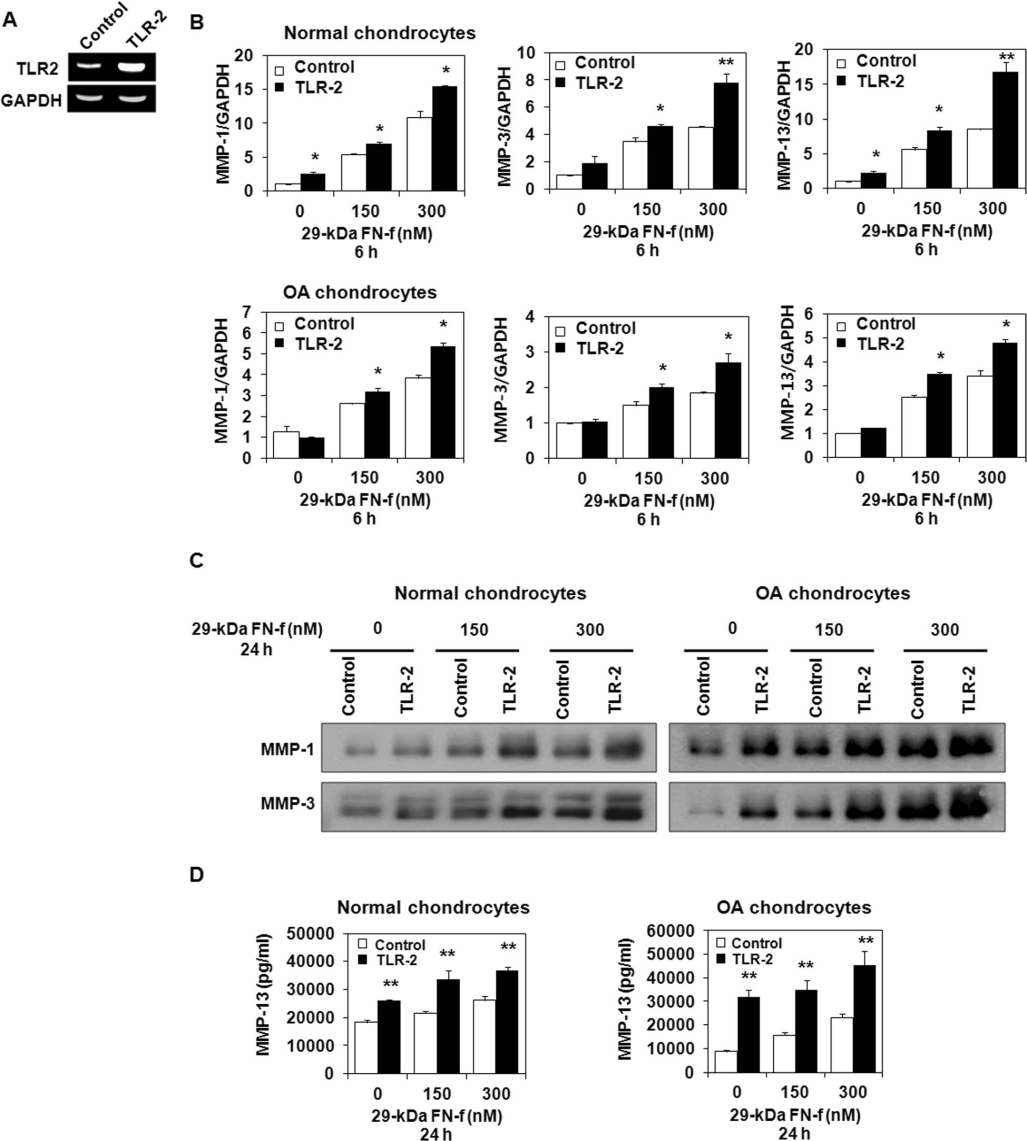
Increased Toll-like receptor (TLR)-2 expression promotes 29-kDa amino-terminal fibronectin fragment (29-kDa FN-f)-induced matrix metalloproteinase (MMP) production in normal and osteoarthritis (OA) chondrocytes. Human chondrocytes were transfected using a TLR-2 expression vector or empty vector as a control. **a** TLR-2 expression was upregulated in chondrocytes transfected with TLR-2 expression vectors. **b** TLR-2 overexpression significantly increased MMP-1, MMP-3, and MMP-13 mRNA expression compared with cells transfected with empty vector. The mRNA expression level in untreated control small interfering RNA transfected chondrocytes was set as 1. Data represent the mean ± SD for triplicate experiments from three different donors. *P < 0.05, **P < 0.01, and ***P < 0.001 vs*.* control. **c** The 29-kDa FN-f-induced release of MMP-1 and MMP-3 was analyzed using Western blot analysis. TLR-2 overexpression enhanced the synthesis of MMP-1 and MMP-3 in normal and OA chondrocytes. **d** MMP-13 production in culture supernatants was determined by enzyme-linked immunosorbent assay. The 29-kDa FN-f-induced MMP-13 production was significantly higher in cells transfected with the TLR-2 expression vector. The bars represent the mean ± SD of triplicate samples from three different donors. *P < 0.05 and **P < 0.01 vs*.* empty vector-transfected chondrocytes

Although it has been reported that FN-fs play important roles in the catabolic responses of human chondrocytes, the precise mechanism remains unclear. To investigate the involvement of TLR-2 in 29-kDa FN-f-mediated MMP production in human chondrocytes, we knocked down TLR-2 in these cells using siTLR-2. RT-PCR analysis revealed significant inhibition of TLR-2 expression (80 %) after the introduction of siTLR-2 (Fig. 2a). In normal and OA chondrocytes, 29-kDa FN-f-induced mRNA expression levels of MMP-1, MMP-3, and MMP-13 were reduced significantly by siTLR-2 (Fig. 2b). Consistent with mRNA expression pattern, 29-kDa FN-f-mediated protein production of these MMPs was also significantly suppressed by siTLR-2 treatment (Fig. 2c and d). In contrast, TLR-2 overexpression by transfection with TLR-2 expression vector (Fig. 3a) resulted in significant upregulation of the 29-kDa FN-f-induced mRNA expression of these MMPs compared with mock vector-transfected chondrocytes (Fig. 3b). Likewise, 29-kDa FN-f-induced MMP protein synthesis was enhanced significantly in TLR-2-overexpressing cells (Fig. 3c and d). These data indicate that TLR-2 is involved in 29-kDa FN-f-stimulated MMP production in human chondrocytes.

### Regulatory effects of TLR-2 on 29-kDa FN-f-mediated catabolic signaling pathways in human articular chondrocytes

**Fig. 4:**
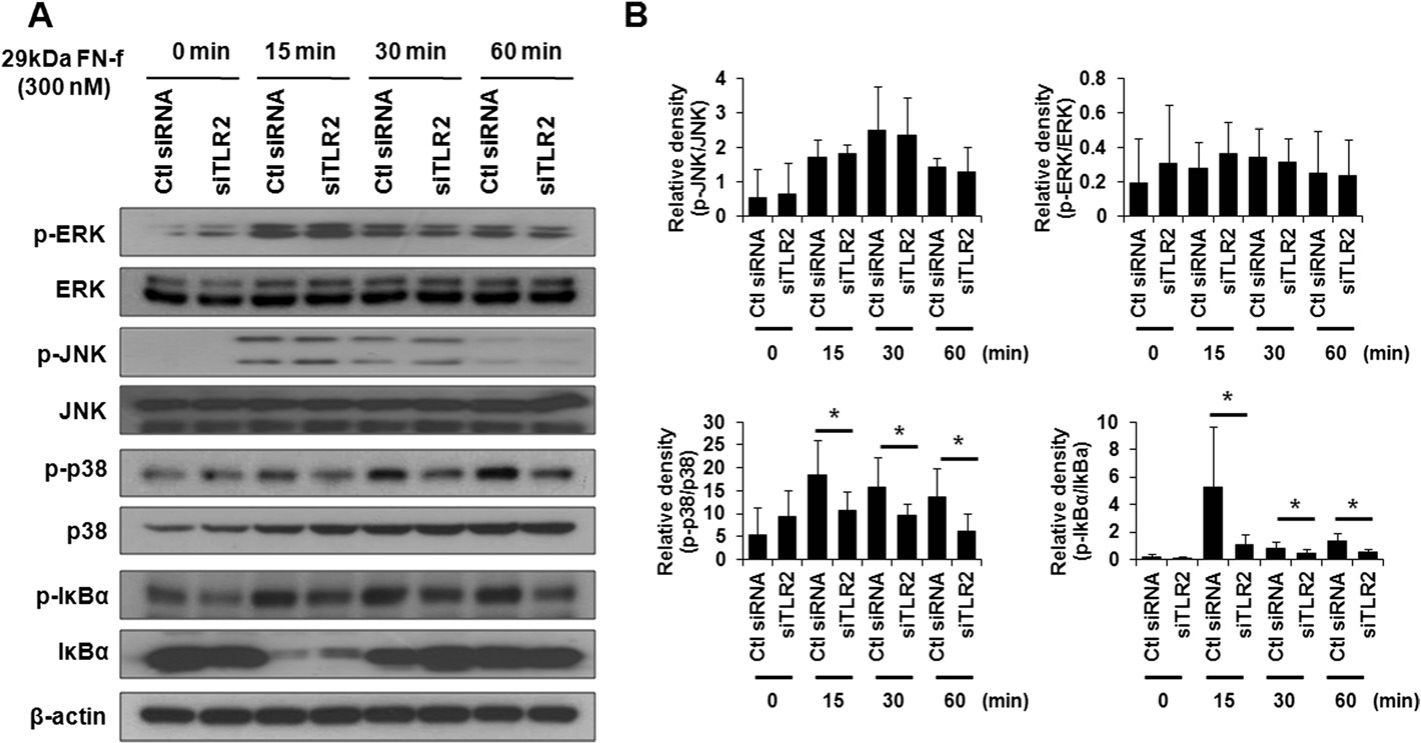
Small interfering Toll-like receptor 2 (siTLR-2) knockdown of endogenous TLR-2 expression inhibits 29-kDa amino-terminal fibronectin fragment (29-kDa FN-f)-mediated catabolic pathways in human chondrocytes. **a** To investigate the effect of TLR-2 on 29-kDa FN-f-induced signal pathways, human chondrocytes were transfected using control small interfering RNA (siRNA) or siTLR-2. After transfection, the cells were stimulated using 29-kDa FN-f for the indicated times, and the levels of phosphorylated nuclear factor of kappa light polypeptide gene enhancer in B-cells inhibitor, alpha (p-IκBα), phosphorylated c-Jun N-terminal kinase (p-JNK), phosphorylated p38 (p-p38), and phosphorylated extracellular signal-regulated kinase (p-ERK) were determined using Western blot analysis. Western blot data are representative of three independent experiments from different donors with similar results. **b** The relative phosphorylation level of JNK, ERK, p38, and IκBα proteins. Protein density was normalized to the respective unphosphorylated proteins. The bars represent the mean ± SD of three independent experiments from different donors with similar results. *P < 0.05 and **P < 0.01 vs*.* control siRNA-transfected chondrocytes. Ctl, control

To determine whether TLR-2 regulates 29-kDa FN-f-mediated signaling pathways, human chondrocytes were transfected with siTLR-2 or control siRNA. Forty-eight hours after transfection, cells were starved using serum-free media for 24 h and then treated with 29-kDa FN-f for the indicated time periods, and the regulatory effects of TLR-2 on 29-kDa FN-f-induced signaling pathways were determined using Western blot analysis. Interestingly, the phosphorylation of IκBα and p38 was apparently inhibited in siTLR-2-transfected cells compared with control siRNA-transfected cells (Fig. 4a and b). In contrast, the phosphorylation of JNK and ERK1/2 was not affected by siTLR-2 in human chondrocytes (Fig. 4a and b). These data indicate that the 29-kDa FN-f-driven activation of the nuclear factor kappa-light-chain-enhancer of activated B cells (NF-κB) and p38 signaling pathways may be associated with TLR-2 in human chondrocytes.

### 29-kDa FN-f upregulates MMP secretion via a MyD88-dependent TLR-2 signaling pathway in human articular chondrocytes

**Fig. 5:**
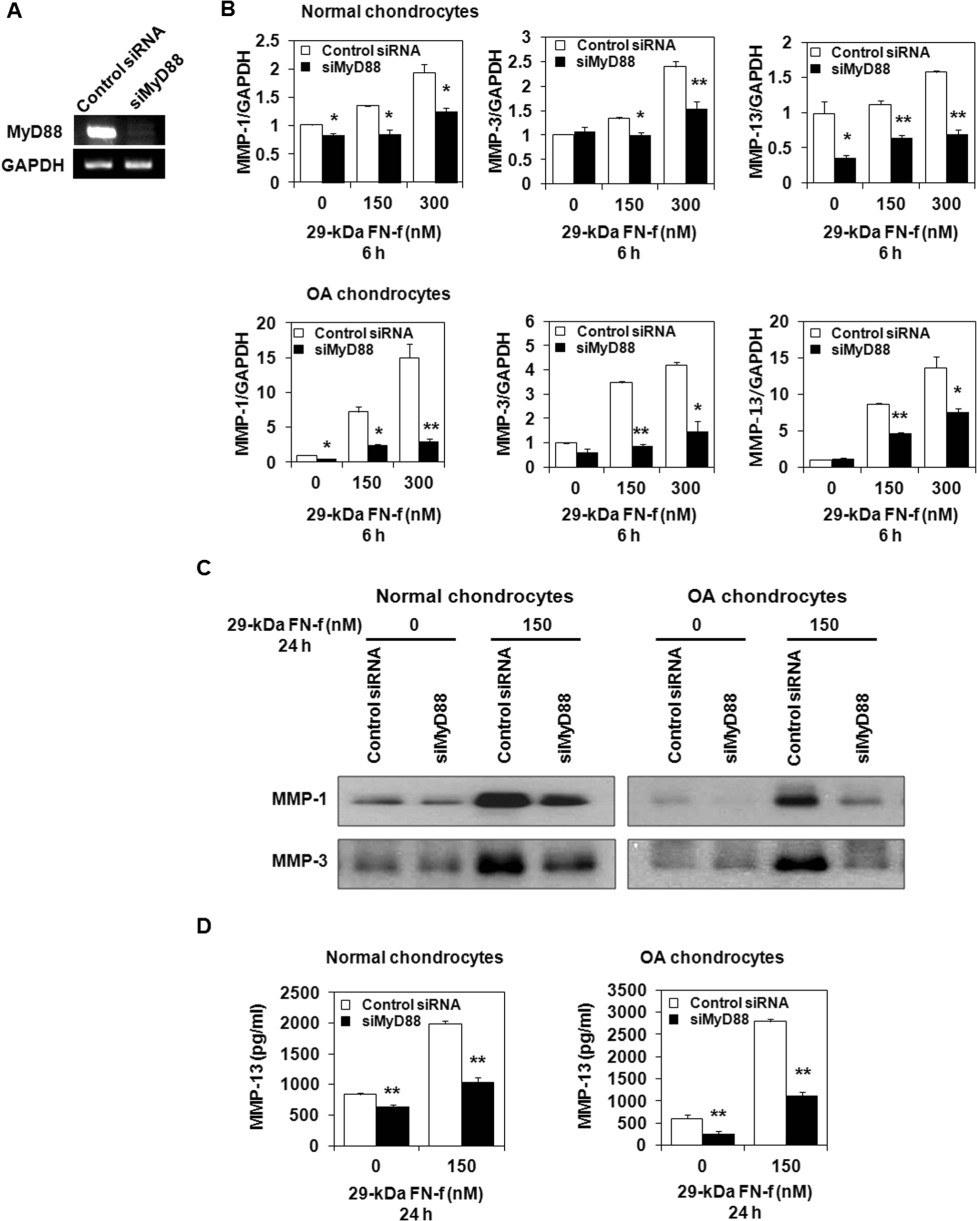
Myeloid differentiation factor 88 (MyD88)-dependent Toll-like receptor (TLR)-2 signaling pathway regulates 29-kDa amino-terminal fibronectin fragment (29-kDa FN-f)-induced matrix metalloproteinase (MMP) production in normal and osteoarthritis (OA) chondrocytes. To determine the involvement of the MyD88-dependent TLR-2 signaling pathway in 29-kDa FN-f-triggered catabolic responses, human chondrocytes were transfected using control small interfering RNA (siRNA) or small interfering MyD88 (siMyD88). After 48 h, the cells were stimulated using 29-kDa FN-f for 6 or 24 h. **a** Inhibition of MyD88 expression by siMyD88. **b** Effect of siMyD88 on mRNA expression of 29-kDa FN-f-induced MMPs was determined using SYBR Green real-time reverse transcription polymerase chain reaction. Glyceraldehyde 3-phosphate dehydrogenase (GAPDH) was used as an internal control. In normal and OA chondrocytes, the expression of MMP-1, MMP-3, and MMP-13 mRNA were reduced significantly by siMyD88 treatment. The mRNA expression level in untreated control siRNA transfected chondrocytes was set as 1. Data represent the mean ± SD for triplicate experiments from three different donors. *P < 0.05, **P < 0.01, and ***P < 0.001 vs*.* control. **c** MMP-1 and MMP-3 protein levels in culture supernatants were determined using Western blot analysis. **d** Enzyme-linked immunosorbent assay showed that 29-kDa FN-f-triggered MMP-13 production was suppressed significantly in siMyD88-transfected cells compared with siRNA-transfected control cells. Values represent the mean ± SD of triplicate samples from three different donors. *P < 0.05 and **P < 0.01 vs*.* control siRNA-transfected chondrocytes

To further elucidate the molecular mechanism underlying the regulatory effect of TLR-2 on 29-kDa FN-f-stimulated MMP production, we transfected human articular chondrocytes with siMyD88 or control siRNA and stimulated them with 29-kDa FN-f. MyD88 siRNA transfection decreased MyD88 mRNA expression in chondrocytes (Fig. 5a). The MMP-1, MMP-3, and MMP-13 mRNA expression levels were significantly reduced in siMyD88-transfected cells compared with the control siRNA-transfected cells (Fig. 5b). In addition, MyD88 knockdown significantly reduced 29-kDa FN-f-induced MMP-1 and MMP-3 protein secretion as well as MMP-13 secretion, as demonstrated using Western blot analysis and ELISA, respectively (Fig. 5c and d). These results suggest that 29-kDa FN-f-induced MMP production is regulated by a MyD88-dependent TLR-2 signaling pathway in human chondrocytes.

### TLR-2 knockdown suppresses OA synovial fluid-induced MMPs production

**Fig. 6:**
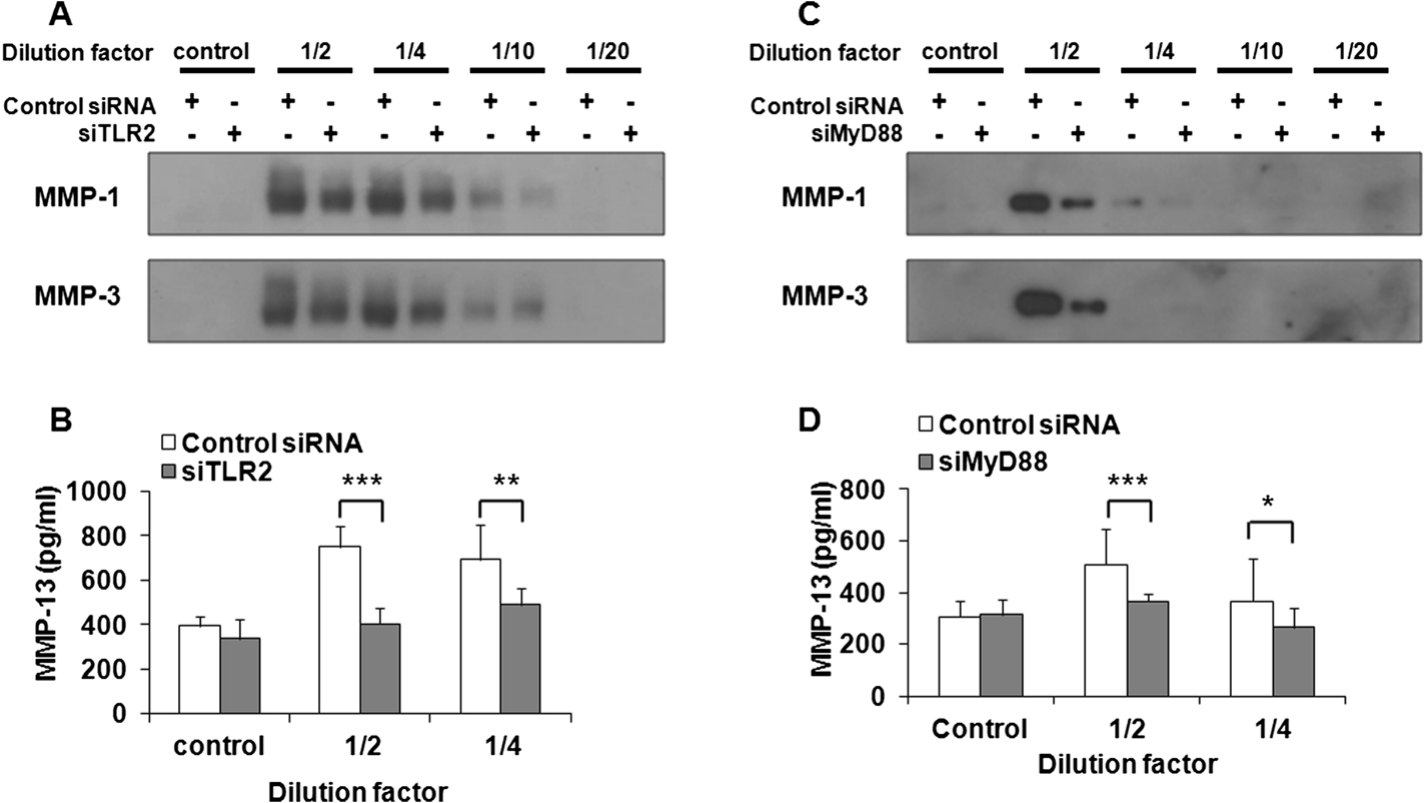
Synovial fluid-induced matrix metalloproteinase (MMP) expression was suppressed by Toll-like receptor (TLR)-2 and myeloid differentiation factor 88 (MyD88) knockdown. **a** TLR-2 in chondrocytes was knocked down by small interfering TLR-2 (siTLR-2) transfection, after which cells were treated with sequentially diluted synovial fluid. MMP-1 and MMP-3 expression levels in culture media were determined using Western blot analysis. **b** The expression levels of MMP-13 in culture media were determined by enzyme-linked immunosorbent assay (ELISA). Data represent the means ± SD for triplicate experiments from three different donors. **c** MyD88 in chondrocytes was knocked down by small interfering MyD88 (siMyD88) transfection, after which cells were treated with sequentially diluted synovial fluid. MMP-1 and MMP-3 expression levels in culture media were determined using Western blot analysis. **d** The expression levels of MMP-13 in culture media were determined using ELISA. *Control* untreated with synovial fluid, *control siRNA* control small interfering RNA-transfected cells, *siTLR-2* small interfering TLR-2 RNA-transfected cells, *siMyD88* small interfering MyD88 RNA-transfected cells. Data represent the mean ± SD for triplicate experiments from three different donors. *P < 0.05, **P < 0.01, and ***P < 0.001 vs*.* control siRNA-transfected chondrocytes

To determine whether the SF of patients with OA induces MMP production through TLR-2, we transfected human chondrocytes using siTLR-2, siMyD88, or control siRNA, followed by treatment with SF serially diluted using serum-free media. The diluted SF had little effect on chondrocyte viability (data not shown). Western blot analysis (Fig. 6a and c) and ELISA (Fig. 6b and d) showed that SF at up to 1:10 dilution upregulated MMP-1, MMP-3, and MMP-13 synthesis in the control siRNA-transfected cells. However, TLR-2 and MyD88 knockdown significantly suppressed SF-induced MMP synthesis (Fig. 6a–d). We could not detect MMP-1, MMP-3, or MMP-13 in SF-exposed media in the absence of chondrocytes (see Additional file 2A and B). We examined whether FN-fs in SF mediate MMP expression in chondrocytes by neutralizing FN-fs with an antibody against FN. Anti-FN antibody significantly reduced SF-induced MMP release from chondrocytes (Additional file 2C and D), meaning that FN or FN-fs in SF attach to receptors, including TLR-2, on chondrocytes and subsequently induce MMPs expression. These data showed that OA SF, which contains various DAMP species, at least in part upregulates MMP production via a TLR-2-mediated signaling pathway.

## Discussion

In this study, we demonstrated that TLR-2, TLR-3, TLR-4, and TLR-5, which play an important role in innate immunity, are more abundantly expressed in OA cartilage than in normal cartilage. Among the different FN-fs investigated, 29-kDa FN-f upregulated TLR-2 expression most potently. 29-kDa FN-f upregulated MMP-1, MMP-3, and MMP-13 expression in chondrocytes via a MyD88-dependent TLR-2 signaling pathway.

FN, an important component of articular cartilage matrix, is composed of a series of homologous domains that form an extended flexible molecule that is extremely sensitive to proteolytic digestion [19]. FN-fs, generated by proteolytic cleavage of FN, are found at elevated levels in OA cartilage and SF [20–22], consequently inducing catabolic mediators and the degradation of cartilage collagen [11]. The 29-kDa, 50-kDa, and 140-kDa FN-fs, which have been studied the most extensively, have a different affinity for cartilage matrix molecules. 29-kDa FN-f shows affinity for heparin, 50-kDa FN-f for gelatin and collagen, and 140-kDa FN-f for heparin and the protein core of heparan sulfate proteoglycan [23]. Although each FN-f may possess specific proteolytic activities, FN-f-induced cartilage catabolism results mostly from increased MMP expression and activity [24, 25]. 29-kDa FN-f was reported to be the most potent inducer of cartilage catabolism, which was corroborated by an in vivo study showing that 29-kDa FN-f injection into rabbit knee joints caused up to 70 % loss of total cartilage proteoglycan within 7 days [26]. In addition to MMPs, 29-kDa FN-f was shown to induce procatabolic cytokines, including interleukin (IL)-1α, IL-1β, IL-6, and tumor necrosis factor (TNF)-α, as well as NO production, in human and bovine cartilage [27].

FN is known to bind several receptors of the integrin family [22, 28], and 120-kDa FN-f, which contains the cell-binding RGD sequence, may bind to and stimulate the α_5_β_1_ integrin receptor, subsequently increasing cytokine and chemokine expression through p38 or JNK mitogen-activated protein kinase (MAPK) activation and the NF-κB-dependent pathway [29, 30]. On one hand, the use of RGD analogues resembling the integrin binding sequence suggested that 29-kDa and 50-kDa FN-fs exert their effects independently of integrin interactions [31]. On the other hand, it was shown that FN-fs, including 29, 50, and 140 kDa, were cross-linked to the integrin receptor subunit α_5_, and the presence of an antisense oligonucleotide to the α_5_ subunit of integrin partially reversed the ability of the FN-fs to suppress proteoglycan synthesis in cartilage explants and high-density chondrocyte cultures [32]. A subject of our further investigation is to see whether α_5_ knockdown has a synergistic effect in the TLR-2 mediation of FN-f response.

Products of cartilage matrix degradation, including FN-fs, hyaluronan fragments, and collagen hydrolysates, are known to play a role as DAMPs and may form a vicious cycle for accelerating cartilage matrix damage [11, 14, 21, 27, 33]. Considerable evidence from many studies suggests that the cellular level and expression of different TLRs could be related to the homeostasis of cartilage matrix. TLRs in articular cartilage and cultured chondrocytes are known to be increased in OA [34, 35]. Furthermore, TLR-2 expression is upregulated by catabolic cytokines, including IL-1β, and TLR-2 ligands, including 45-kDa and 29-kDa FN-f, which strongly induce catabolic responses in chondrocytes [2, 35]. In addition, TLR-2 can recognize endogenous ligands, including monosodium urate crystal [35] and hyaluronan fragments [14].

In the present study, we demonstrated a strong upregulation of TLR-2 by 29-kDa FN-f and a relatively weak but significant increase by 45-kDa FN-f, as well as upregulation of TLR-2 in OA cartilage compared with normal cartilage, suggesting a relationship between catabolic signaling induced by FN-fs and TLR-2. Hyaluronan fragments increased proinflammatory cytokines by activating CD44 and TLR-4 in chondrocytes, indicating a role for DAMP recognition by TLR in cartilage damage progression [33]. In addition, MyD88-dependent TLR-2/TLR-4 signaling was demonstrated to be essential for procatabolic responses to low molecular weight hyaluronan and high-mobility group box chromosomal protein 1 [14]. In agreement with previous studies, we found that 29-kDa FN-f significantly activated procatabolic responses in human articular chondrocytes, which was mediated through TLR-2. In contrast, knocking down TLR-4 did not have a significant influence on the 29-kDa FN-f-mediated catabolic response (data not shown). Although the unavailability of suitable antibodies and technical challenges prevented us from confirming the direct binding of FN-fs to chondrocyte TLR-2 in the immunoprecipitation assay, the interaction of fluorescently labeled 29-kDa FN-f with chondrocytes was abolished by using excess TLR-2 antibody, suggesting a direct interaction (data not shown).

Downstream signaling pathways of TLRs include MyD88-dependent and MyD88-independent pathways [13]. MyD88, a Toll/IL-1 receptor (TIR) adaptor, modulates TLR signaling pathways, with the exception of the TLR-3 and MyD88-independent TLR-4 signaling pathways [36]. Upon stimulation, activated TLR-2 transiently forms a complex with MyD88 via interaction between individual TIR domains. In addition, IL-1 receptor-associated kinase and TNF receptor-associated factor 6 are recruited to the receptor signaling complex, finally activating the MAPK and transcription regulators, including activator protein 1 and NF-κB, the latter mediating the expression of several proinflammatory cytokines and other inflammatory molecules [13, 37]. We found that knocking down TLR-2 significantly suppressed downstream signaling pathways mediated by FN-fs, including NF-κB and p38. In addition, knocking down MyD88 uniformly inhibited MMP upregulation mediated by FN-fs. Previous studies demonstrated that FN-f containing the RGD cell-binding domain stimulated small GTPase Rac1 signaling in addition to MAPK and NF-κB signaling pathways in human chondrocytes, which were involved in MMP-13 expression [20, 29]. Whether TLR-2 mediates pathways regulated by other FN-fs is the subject of further research.

It was reported previously that factors produced by the synovium may stimulate OA chondrocytes to express catabolic mediators. For example, OA SF significantly upregulated p55 TNF receptor expression in chondrocytes compared with normal serum [38]. Chondrocyte death was observed after exposure of chondrocytes to RA SF but not to OA SF, and active secretion of cytokines, including vascular endothelial growth factor, by chondrocytes treated with OA SF was observed [39], suggesting a role for OA SF in the stimulation of a catabolic response in OA. In addition to the cellular elements of the synovium, it is plausible that OA SF also contains cartilage degradation products. We demonstrated that OA SF increased MMP expression in OA chondrocytes, which was mediated partly via TLR-2. Neutralizing antibody against FN significantly reduced SF-induced MMP release from chondrocytes, meaning that FN or FN-fs in SF attach to receptors, including TLR-2, on chondrocytes and subsequently induce MMPs. This result is compatible with our hypothesis that DAMP contained in SF may at least in part lead to cartilage degradation via TLR-2.

## Conclusions

Our results demonstrate that 29-kDa FN-f may induce catabolic responses, including MMP expression, by activation of NF-κB and p38 through an MyD88-dependent TLR-2 signaling pathway in articular chondrocyte culture (see Additional file 3). DAMP contained in SF may lead at least in part to cartilage degradation via TLR-2. Our study therefore provides additional evidence that the TLR-2-mediated signaling pathway may contribute to cartilage matrix degradation in OA and thus could serve as a useful therapeutic target for OA.

## Additional files

Additional file 1:
**TLR-1, TLR-3, TLR-4, and TLR-5 mRNA expression levels in OA chondrocytes were measured at 6 h after treatment with intact FN or various FN-fs.** The expression level of TLR family members was described as raw *C*_t_ values as well as relative values normalized to GAPDH. Data represent the mean ± SD for triplicate experiments from three different donors (n = 3). A significant increase (**P* < 0.05) or a significant decrease (^#^ *P* < 0.05, ^##^ *P* < 0.001) vs. control. (PPTX 69 kb) Additional file 2:
**The release of MMP-1, MMP-3, and MMP-13 into culture medium in SF-treated chondrocytes.** Chondrocytes were incubated with sequentially diluted SF for 24 h, washed twice with serum-free DMEM, and incubated in serum-free DMEM for 24 h. Also, control medium was prepared by incubating with diluted SF only (without chondrocytes), washing with serum-free DMEM, and incubating in serum-free DMEM for 24 h. Then the medium was collected for analysis of MMP-1, MMP-3, and MMP-13 release into culture medium. **A** The levels of MMP-1 and MMP-3 in culture media were determined using Western blot analysis. *C* control, untreated with SF. **B** The expression levels of MMP-13 in culture media were determined using ELISA. Data represent the mean ± SD for triplicate experiments from three different donors. **P* < 0.05 vs*.* control treated with SF in the absence of chondrocytes. **C** and **D** Release of MMP-1, MMP-3, and MMP-13 induced by SF was reduced by neutralization of SF with neutralizing antibody (FN antibody). SF was neutralized with diluted neutralizing antibody [fibronectin antibody (N-20), sc-6953] against FN for 6 h. Chondrocytes were exposed for 24 h to the neutralized SF, washed twice with serum-free DMEM, and incubated in serum-free DMEM for additional 24 h. Then the medium was collected for analysis of MMP-1, MMP-3, and MMP-13 release into the culture medium. *SF* 1:2 diluted synovial fluid. Dilution factor of neutralization antibody, 1:4, 1:8, and 1:16. **C** The levels of MMP-1 and MMP-3 in culture media were determined using Western blot analysis. **D** Release of MMP-13 in the culture media was determined using ELISA. Data represent the mean ± SD for triplicate experiments from three different donors. ^#^ *P* < 0.05, ^##^ *P* < 0.01, and ^###^ *P* < 0.001 vs*.* untreated control. ****P* < 0.001 vs*.* SF-treated cells. (PPTX 127 kb) Additional file 3:
**Schematic signaling pathway through which 29 kDa FN-f mediated MMP expression via TLR-2.** (PPTX 40 kb)

## Abbreviations

29-kDa FN-f: 29-kDa amino-terminal fibronectin fragment
*C*_t_: Cycle threshold
DAMP: Damage-associated molecular pattern
DMEM: Dulbecco’s modified Eagle’s medium
ECM: Extracellular matrix
ELISA: Enzyme-linked immunosorbent assay
ERK: Extracellular signal-regulated kinase
FN-f: Fibronectin fragment
GAPDH: Glyceraldehyde 3-phosphate dehydrogenase
IκBα: nuclear factor of kappa light polypeptide gene enhancer in B-cells inhibitor, alpha
IL: Interleukin
LPS: Lipopolysaccharide
MAPK: Mitogen-activated protein kinase
MMP: Matrix metalloproteinase
MyD88: Myeloid differentiation factor 88
NF-κB: Nuclear factor kappa-light-chain-enhancer of activated B cells
NO: Nitric oxide
OA: Osteoarthritis
PGN: Peptidoglycan
p-JNK: Phosphorylated c-Jun N-terminal kinase
qRT-PCR: quantitative real-time reverse transcription polymerase chain reaction
RA: Rheumatoid arthritis
SD: Standard deviation
SDS: Sodium dodecyl sulfate
SF: Synovial fluid
siMyD88: Small interfering myeloid differentiation factor 88
siRNA: Small interfering RNA
siTLR-2: Small interfering Toll-like receptor 2
TIR: Toll/interleukin-1 receptor
TLR: Toll-like receptor
TNF: tumor necrosis factor
