# Supplementary material for: Fibronectin fragment-induced expression of matrix metalloproteinases is mediated by MyD88-dependent TLR-2 signaling pathway in human chondrocytes
Source: Arthritis Res Ther. 2015 Nov 12;17:320. doi: 10.1186/s13075-015-0833-9 (PMC4643537; doi:10.1186/s13075-015-0833-9)
Supplement: Additional file 1: — TLR-1, TLR-3, TLR-4, and TLR-5 mRNA expression levels in OA chondrocytes were measured at 6 h after treatment with intact FN or various FN-fs. The expression level of TLR family members was described as raw C t values as well as relative values normalized to GAPDH. Data represent the mean ± SD for triplicate experiments from three different donors (n = 3). A significant increase (*P < 0.05) or a significant decrease (# P < 0.05, ## P < 0.001) vs. control. (PPTX 69 kb) [file 13075_2015_833_MOESM1_ESM.pptx]

## Slide 1
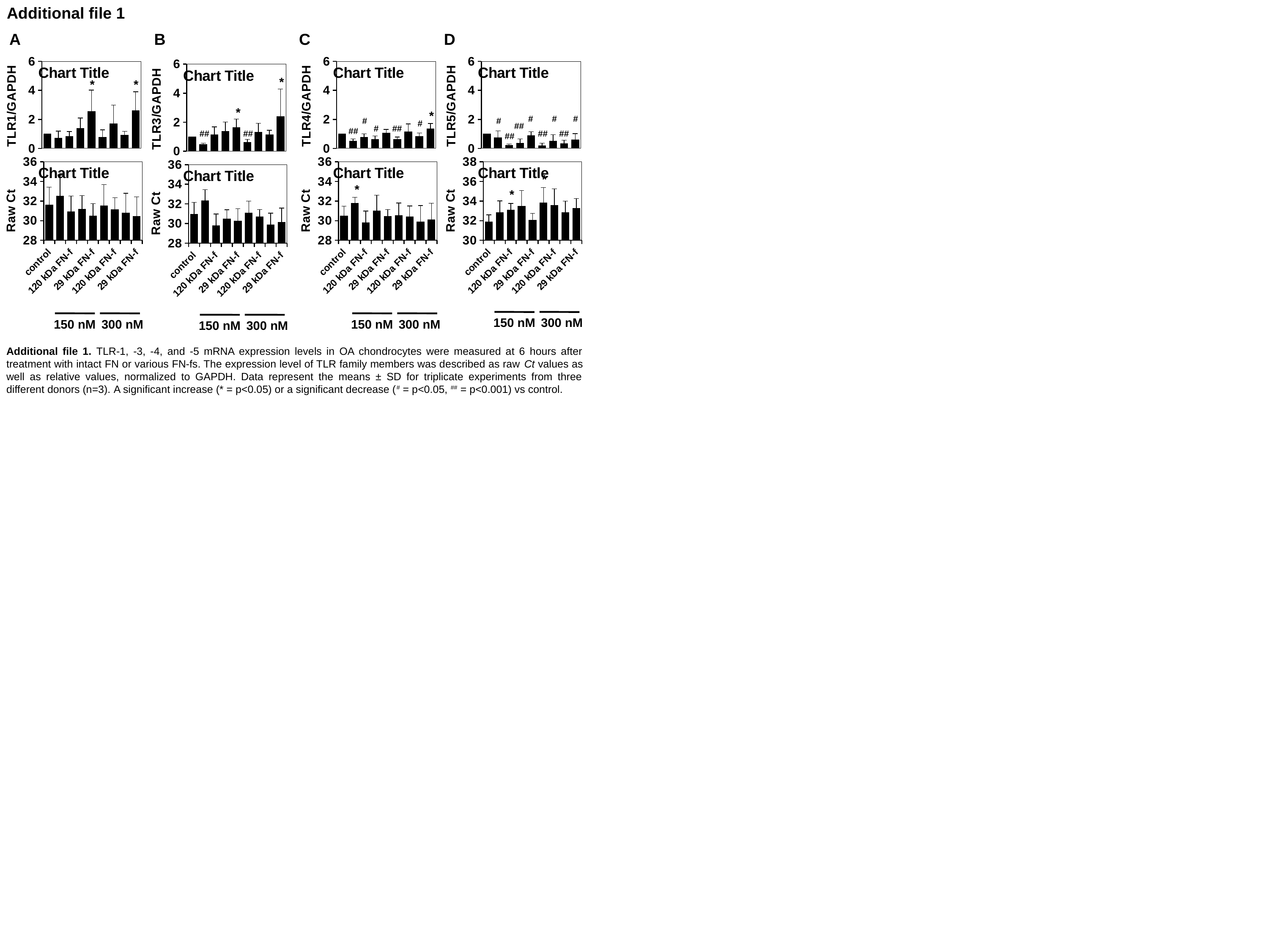

Additional file 1
B
D
A
C
### Chart:
| Category | |
|---|---|
| control | 1.0 |
| FN | 0.7257156901160526 |
| 120 kDa FN-f | 0.8175479711880806 |
| 45 kDa FN-f | 1.3682364866931465 |
| 29 kDa FN-f | 2.5482663455041314 |
| FN | 0.7670929670168737 |
| 120 kDa FN-f | 1.7054231126805246 |
| 45 kDa FN-f | 0.929814016716574 |
| 29 kDa FN-f | 2.593928876896085 |
### Chart:
| Category | |
|---|---|
| control | 1.0 |
| FN | 0.49476978972792013 |
| 120 kDa FN-f | 0.7799066450718629 |
| 45 kDa FN-f | 0.6371970774009326 |
| 29 kDa FN-f | 1.0501715174689006 |
| FN | 0.6130848800531071 |
| 120 kDa FN-f | 1.1614707369513728 |
| 45 kDa FN-f | 0.8151607297744206 |
| 29 kDa FN-f | 1.3400418418502058 |
### Chart:
| Category | |
|---|---|
| control | 1.0 |
| FN | 0.748233130055035 |
| 120 kDa FN-f | 0.2111651939171194 |
| 45 kDa FN-f | 0.36714000657759294 |
| 29 kDa FN-f | 0.8969291366493483 |
| FN | 0.1946937187866907 |
| 120 kDa FN-f | 0.5066125908896936 |
| 45 kDa FN-f | 0.34381268211422056 |
| 29 kDa FN-f | 0.584965929566243 |
### Chart:
| Category | |
|---|---|
| control | 1.0 |
| FN | 0.45832504563710647 |
| 120 kDa FN-f | 1.153829184177752 |
| 45 kDa FN-f | 1.359821618597319 |
| 29 kDa FN-f | 1.648280805416057 |
| FN | 0.6037420797625159 |
| 120 kDa FN-f | 1.3211376519278113 |
| 45 kDa FN-f | 1.1405883146437608 |
| 29 kDa FN-f | 2.3949717731714752 |*
*
*
*
*
#
#
#
#
#
#
##
#
##
##
##
##
##
##
##
### Chart:
| Category | |
|---|---|
| control | 31.615673065185543 |
| FN | 32.52290821075449 |
| 120 kDa FN-f | 30.89897187550861 |
| 45 kDa FN-f | 31.186669985453232 |
| 29 kDa FN-f | 30.503701845804827 |
| FN | 31.538246790567985 |
| 120 kDa FN-f | 31.15240764617919 |
| 45 kDa FN-f | 30.79855060577387 |
| 29 kDa FN-f | 30.437575976053868 |
### Chart:
| Category | |
|---|---|
| control | 30.500014940897625 |
| FN | 31.77629725138343 |
| 120 kDa FN-f | 29.776773134867284 |
| 45 kDa FN-f | 31.02085018157959 |
| 29 kDa FN-f | 30.430342356363834 |
| FN | 30.532083193461087 |
| 120 kDa FN-f | 30.40511035919184 |
| 45 kDa FN-f | 29.888633410135803 |
| 29 kDa FN-f | 30.119930267333984 |
### Chart:
| Category | |
|---|---|
| control | 31.893842379252096 |
| FN | 32.815333366394015 |
| 120 kDa FN-f | 33.112111091613734 |
| 45 kDa FN-f | 33.47825336456295 |
| 29 kDa FN-f | 32.062120755513476 |
| FN | 33.845546722412074 |
| 120 kDa FN-f | 33.55087820688881 |
| 45 kDa FN-f | 32.82284577687589 |
| 29 kDa FN-f | 33.244074185689186 |
### Chart:
| Category | |
|---|---|
| control | 30.95765844980881 |
| FN | 32.3123191197713 |
| 120 kDa FN-f | 29.786101977030416 |
| 45 kDa FN-f | 30.484316380818672 |
| 29 kDa FN-f | 30.26755777994789 |
| FN | 31.06297842661539 |
| 120 kDa FN-f | 30.703430811564054 |
| 45 kDa FN-f | 29.85032653808588 |
| 29 kDa FN-f | 30.130279858907056 |*
*
*
150 nM
300 nM
150 nM
300 nM
150 nM
300 nM
150 nM
300 nM
Additional file 1. TLR-1, -3, -4, and -5 mRNA expression levels in OA chondrocytes were measured at 6 hours after treatment with intact FN or various FN-fs. The expression level of TLR family members was described as raw Ct values as well as relative values, normalized to GAPDH. Data represent the means ± SD for triplicate experiments from three different donors (n=3). A significant increase (* = p<0.05) or a significant decrease (# = p<0.05, ## = p<0.001) vs control.
