# Supplementary material for: Fibronectin fragment-induced expression of matrix metalloproteinases is mediated by MyD88-dependent TLR-2 signaling pathway in human chondrocytes
Source: Arthritis Res Ther. 2015 Nov 12;17:320. doi: 10.1186/s13075-015-0833-9 (PMC4643537; doi:10.1186/s13075-015-0833-9)
Supplement: Additional file 2: — The release of MMP-1, MMP-3, and MMP-13 into culture medium in SF-treated chondrocytes. Chondrocytes were incubated with sequentially diluted SF for 24 h, washed twice with serum-free DMEM, and incubated in serum-free DMEM for 24 h. Also, control medium was prepared by incubating with diluted SF only (without chondrocytes), washing with serum-free DMEM, and incubating in serum-free DMEM for 24 h. Then the medium was collected for analysis of MMP-1, MMP-3, and MMP-13 release into culture medium. A The levels of MMP-1 and MMP-3 in culture media were determined using Western blot analysis. C control, untreated with SF. B The expression levels of MMP-13 in culture media were determined using ELISA. Data represent the mean ± SD for triplicate experiments from three different donors. *P < 0.05 vs. control treated with SF in the absence of chondrocytes. C and D Release of MMP-1, MMP-3, and MMP-13 induced by SF was reduced by neutralization of SF with neutralizing antibody (FN antibody). SF was neutralized with diluted neutralizing antibody [fibronectin antibody (N-20), sc-6953] against FN for 6 h. Chondrocytes were exposed for 24 h to the neutralized SF, washed twice with serum-free DMEM, and incubated in serum-free DMEM for additional 24 h. Then the medium was collected for analysis of MMP-1, MMP-3, and MMP-13 release into the culture medium. SF 1:2 diluted synovial fluid. Dilution factor of neutralization antibody, 1:4, 1:8, and 1:16. C The levels of MMP-1 and MMP-3 in culture media were determined using Western blot analysis. D Release of MMP-13 in the culture media was determined using ELISA. Data represent the mean ± SD for triplicate experiments from three different donors. # P < 0.05, ## P < 0.01, and ### P < 0.001 vs. untreated control. ***P < 0.001 vs. SF-treated cells. (PPTX 127 kb) [file 13075_2015_833_MOESM2_ESM.pptx]

## Slide 1
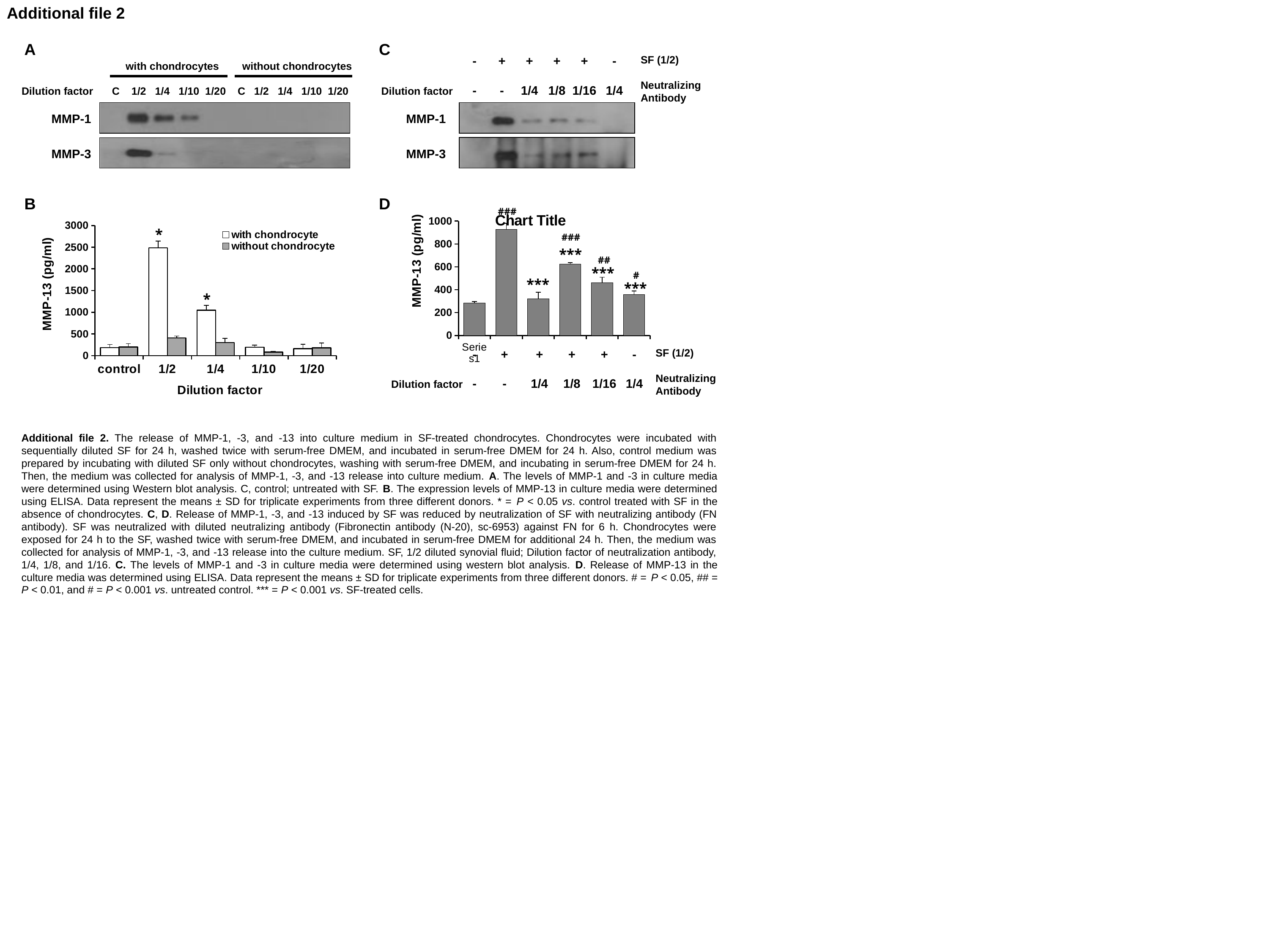

Additional file 2
A
with chondrocytes
without chondrocytes
Dilution factor
 C 1/2 1/4 1/10 1/20 C 1/2 1/4 1/10 1/20
MMP-1
MMP-3
B
### Chart
| Category | with chondrocyte | without chondrocyte |
|---|---|---|
| control | 183.534 | 198.24024999999997 |
| 1/2 | 2485.35625 | 406.4807499999987 |
| 1/4 | 1048.8497499999999 | 301.125174999999 |
| 1/10 | 195.29899999999998 | 81.76675 |
| 1/20 | 161.76875 | 179.41625000000002 |*
*
C
-
-
+
-
+
1/4
+
1/8
+
1/16
-
1/4
SF (1/2)
Neutralizing
Antibody
Dilution factor
MMP-1
MMP-3
D
### Chart:
| Category | |
|---|---|
| | 284.8308799999997 |
| | 928.4702933333332 |
| | 318.7385066666663 |
| | 625.1181333333335 |
| | 460.1211999999995 |
| | 359.30655999999937 |###
###
***
##
***
#
***
***
-
-
+
-
+
1/4
+
1/8
+
1/16
-
1/4
SF (1/2)
Neutralizing
Antibody
Dilution factor
Additional file 2. The release of MMP-1, -3, and -13 into culture medium in SF-treated chondrocytes. Chondrocytes were incubated with sequentially diluted SF for 24 h, washed twice with serum-free DMEM, and incubated in serum-free DMEM for 24 h. Also, control medium was prepared by incubating with diluted SF only without chondrocytes, washing with serum-free DMEM, and incubating in serum-free DMEM for 24 h. Then, the medium was collected for analysis of MMP-1, -3, and -13 release into culture medium. A. The levels of MMP-1 and -3 in culture media were determined using Western blot analysis. C, control; untreated with SF. B. The expression levels of MMP-13 in culture media were determined using ELISA. Data represent the means ± SD for triplicate experiments from three different donors. * = P < 0.05 vs. control treated with SF in the absence of chondrocytes. C, D. Release of MMP-1, -3, and -13 induced by SF was reduced by neutralization of SF with neutralizing antibody (FN antibody). SF was neutralized with diluted neutralizing antibody (Fibronectin antibody (N-20), sc-6953) against FN for 6 h. Chondrocytes were exposed for 24 h to the SF, washed twice with serum-free DMEM, and incubated in serum-free DMEM for additional 24 h. Then, the medium was collected for analysis of MMP-1, -3, and -13 release into the culture medium. SF, 1/2 diluted synovial fluid; Dilution factor of neutralization antibody, 1/4, 1/8, and 1/16. C. The levels of MMP-1 and -3 in culture media were determined using western blot analysis. D. Release of MMP-13 in the culture media was determined using ELISA. Data represent the means ± SD for triplicate experiments from three different donors. # = P < 0.05, ## = P < 0.01, and # = P < 0.001 vs. untreated control. *** = P < 0.001 vs. SF-treated cells.
