# Supplementary material for: Fibronectin fragment-induced expression of matrix metalloproteinases is mediated by MyD88-dependent TLR-2 signaling pathway in human chondrocytes
Source: Arthritis Res Ther. 2015 Nov 12;17:320. doi: 10.1186/s13075-015-0833-9 (PMC4643537; doi:10.1186/s13075-015-0833-9)
Supplement: Additional file 3: — Schematic signaling pathway through which 29 kDa FN-f mediated MMP expression via TLR-2. (PPTX 40 kb) [file 13075_2015_833_MOESM3_ESM.pptx]

## Slide 1
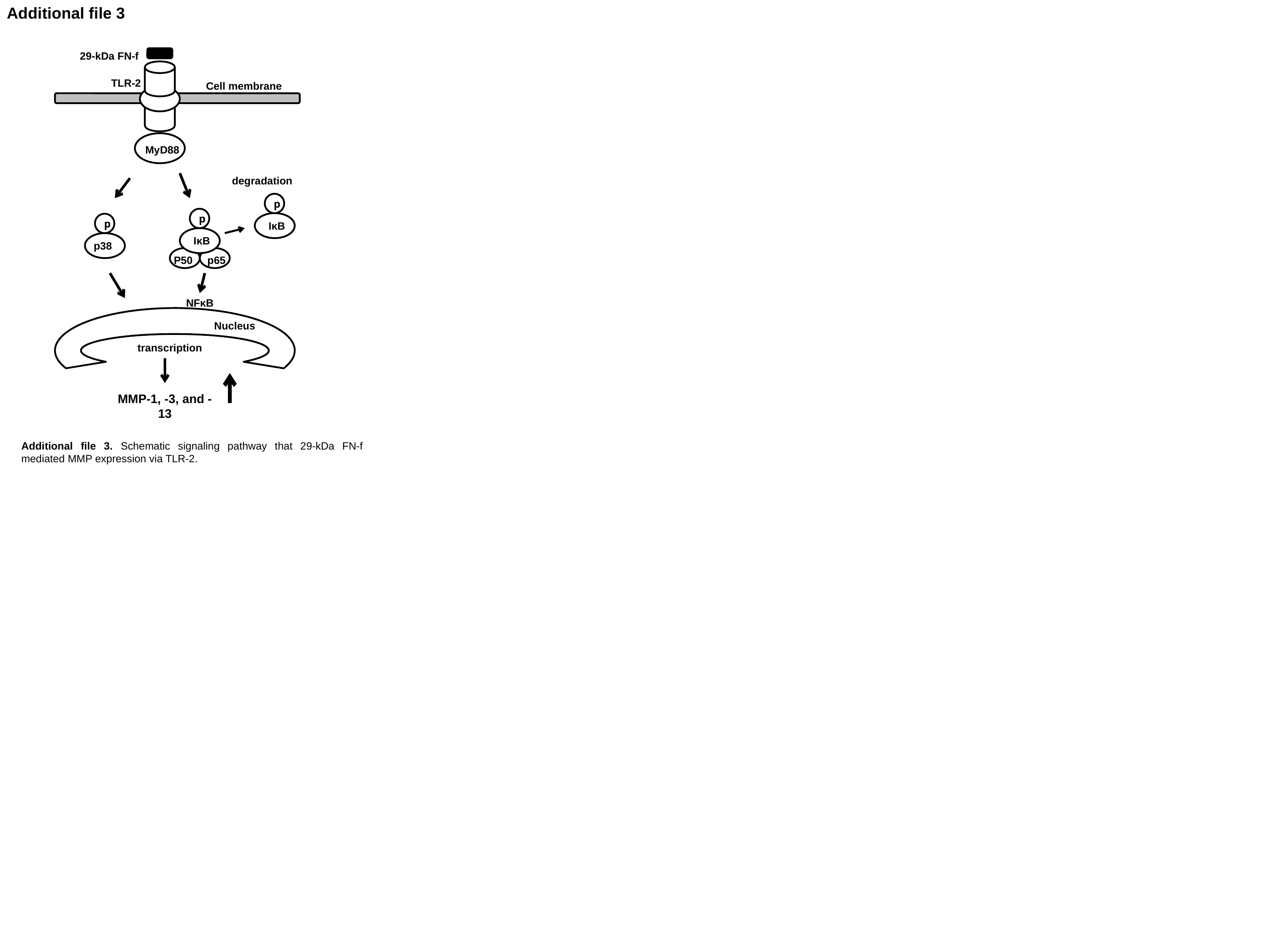

Additional file 3
29-kDa FN-f
TLR-2
Cell membrane
MyD88
degradation
p
IκB
p
IκB
p
p38
P50 p65
NFκB
Nucleus
transcription
MMP-1, -3, and -13
Additional file 3. Schematic signaling pathway that 29-kDa FN-f mediated MMP expression via TLR-2.
